# Urinary Incontinence in Adulthood in a Course of Ectopic Ureter—Description of Two Clinical Cases with Review of Literature

**DOI:** 10.3390/ijerph18137084

**Published:** 2021-07-02

**Authors:** Iga Kuliniec, Przemysław Mitura, Paweł Płaza, Damian Widz, Damian Sudoł, Michał Godzisz, Aleksandra Kołodyńska, Marta Monist, Agata Wisz, Krzysztof Bar

**Affiliations:** 1 Department of Urology and Oncological Urology, Medical University of Lublin, Jaczewskiego 8, 20-954 Lublin, Poland; p_mitura@wp.pl (P.M.); pawel.plaza@gmail.com (P.P.); damian.widz@gmail.com (D.W.); eumedon@gmail.com (D.S.); michal.godzisz92@gmail.com (M.G.); krzysztof.bar@umlub.pl (K.B.); 2 2nd Department of Gynecology, Medical University of Lublin, Jaczewskiego 8, 20-954 Lublin, Poland; kolodynska.aleksandra@gmail.com (A.K.); martamonist@wp.pl (M.M.); 3 Department of Diagnostic Imaging, Radiology and Nuclear Medicine, Faculty of Medical Science in Katowice, Medical University of Silesia, Medyków 16, 40-752 Katowice, Poland; agwisz@gmail.com

**Keywords:** duplex kidney, ectopic ureter in adulthood, ureteric enuresis, urinary incontinence

## Abstract

Urinary tract pathologies are the most common congenital abnormalities. Duplex colleting system occurs at different stages of completion and is usually asymptomatic. Ureteral ectopia is an associated anomaly which may manifest as continuous incontinence. The aim of this article is to present two patients with duplex kidney and ureteral ectopia. Both patients presented symptoms of continuous urinary incontinence and became symptomatic in the adult life.

## 1. Introduction

**Figure 1:**
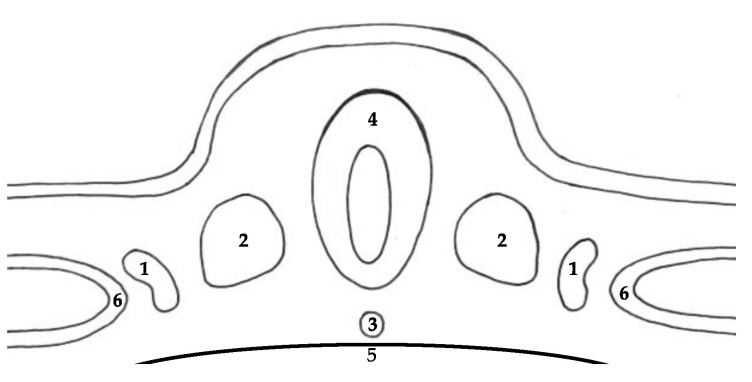
Structures in the dorsal part of the embryo: (1) intermediate mesoderm, (2) paraxial mesoderm, (3) notochord, (4) neural tube, (5) yolk sac, (6) lateral plate.

The development of the urinary system is a highly complex process. It involves several consecutive steps that need to be precisely maintained in order to achieve physiological development. The development of the urinary system takes place between 4th and 10th gestational week [1]. Kidneys and ureters arise from the embryological structure located in the dorsal part of the embryo called intermediate mesoderm (Figure 1) [2].

**Figure 2:**
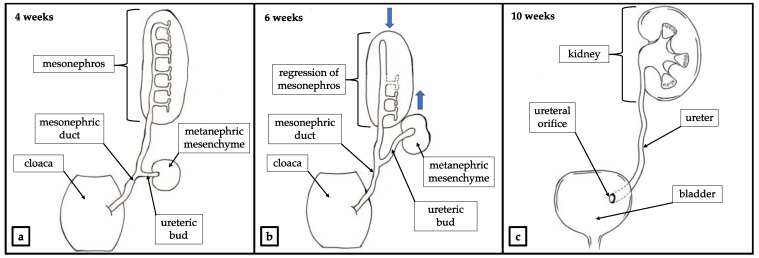
Physiological development of renal system. (**a**) Outgrowth of ureteric bud. (**b**) The metanephric mesenchyme and the ureteric bud migrate cranially toward the final localization of the permanent kidney. Mesonephros atrophies and degenerates. Mesonephric duct migrates caudally and acquires reproductive function [4]. Movement of the structures indicated with blue arrows. (**c**) Developed urinary system.

During the process of embryogenesis, the metanephric mesenchyme and ureteric buds become ureters, renal pelvis, major and minor calyces and collecting tubules. These structures migrate cranially towards the final localization of the permanent kidney (Figure 2) [3].

**Figure 3:**
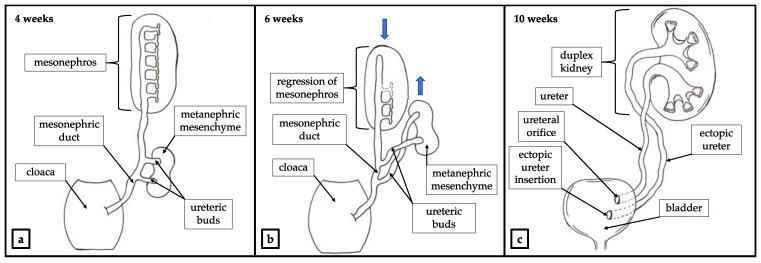
Development of the duplex kidney. (**a**) Outgrowth of two ureteric buds. (**b**) The metanephric mesenchyme and tureteric buds migrate cranially toward the final localization of the permanent kidney in accordance with Weigert—Mayer law. Mesonephros atrophies and degenerates. Mesonephric duct migrates caudally and acquires reproductive function [4]. Movement of the structures indicated with blue arrows. (**c**) Developed duplex kidney with ureteral ectopia.

An abnormal interaction between the metanephric mesenchyme and the ureteric bud may lead to congenital abnormalities of the kidney and urinary tract (CAKUT). Those constitute a wide range of anatomical and histopathological pathologies which include deformation of the urinary tract such as kidney agenesis, polycystic kidneys, horseshoe kidney, duplex collecting system and duplicated ureters [5]. Depending on the kind of abnormal interaction between the metanephric mesenchyme and the ureteric bud different forms of CAKUT may be presented clinically. As a result of abnormal gene signaling in the ureteric bud, a patient may present with duplicated ureter. A complete or incomplete duplication of the ureter with duplex of the kidney may occur as a result of partial or total division of the ureteric bud (Figure 3) [6,7].

Duplex systems may have a broad spectrum of manifestation including ectopic ureter and continuous urinary leakage [8]. Although these pathologies are well known and described in pediatric patients, limited cases have been reported in adults until now [9,10]. We describe two cases with ureteral duplication and ureteric enuresis which became symptomatic in adulthood. These data should be taken into account in the differential diagnosis of urinary incontinence in women.

## 2. Case Reports

### 2.1. Case Study No. 1

In 2006 a 55-year-old female patient was admitted to the Department of Gynecology due to urinary incontinence. She had reported continuous leakage of urine during the night over a period of few years. On daily basis the patient used diaper pants, changing them 3–4 times a day. She gave birth 5 times vaginally and the last menstruation occurred at the age of 48. During the hospitalization, due to complete prolapse of the uterus, a transvaginal hysterectomy without appendages was performed. At the same time, a double TOT mesh was installed. The patient was discharged home in good condition. One month postoperatively the patient was admitted to the hospital due to urgency with urinary leakage. Based on urine analysis the urinary tract infection was diagnosed and an antibiotic therapy was introduced (Furazidinum, Metronidazole, herbal OTC). Diagnostic cystoscopy was normal. The patient was discharged deciding to postpone further treatment and continue using diaper pants.

**Figure 4:**
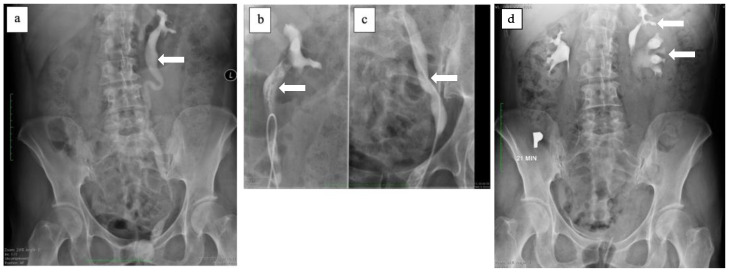
(**a**) Contrasted ectopic ureter (white arrow). (**b**) Contrasted proximal ectopic ureter (white arrow). (**c**) Contrasted distal ectopic ureter (white arrow). (**d**) Contrasted duplex kidney on the left side (white arrows) and normal pelvicalyceal system on the right side.

In 2012 the patient decided to continue the diagnostic process. The gynecological examination was normal, but due to the continuous urine leakage into vagina, it was decided to perform double dye test with methylene blue. During the examination no vesico-vaginal fistula was visualized, however the urine-like discharge was still visible in the vagina. After the urological consultation, it was decided to perform additional testing, such as CT scan and cystoscopy. The CT scan showed a duplex collecting system on the left side with a dilation of the upper moiety ureter. Both CT and diagnostic cystoscopy did not reveal vesico-vaginal fistula. The patient was then admitted to the Department of Urology for further diagnostic and possible treatment. Urography was carried out and revealed ectopic ureter opening into the urethra (Figure 4). Renal scintigraphy was performed and confirmed good function of the upper moiety of the left kidney. The patient was qualified for the surgery and underwent Leadbetter- Politano ureterocystoneostomy. A DJ stent was left in the transplanted ureter. One month after the surgery the patient was admitted to the outpatient clinic of the Department of Urology and the DJ was removed. She confirmed complete continence.

### 2.2. Case Study No. 2

A 36-year-old female patient diagnosed in childhood with asymptomatic bilateral duplex collecting system with bilateral complete ureteric duplication and ectopic ureter of the upper moiety of the left kidney was admitted the Department of Urology for further evaluation because of urinary incontinence.

Since 2007 the patient was diagnosed and treated several times due to bilateral nephrolithiasis, using both URSL and PCNL. The last CT scan was performed in 2014 and confirmed bilateral duplication of collecting system and bilateral complete duplication of ureters, with poorly contrasted upper moiety of the left kidney and its ureter on the left side. The CT scan did not reveal the ectopic ureteral insertion. The patient was asymptomatic until October 2015 when she underwent a cesarean section due to the pelvic position of the fetus. The surgery went well and there was no early postoperative complication. However, during postpartum period, patient reported urinary incontinence symptoms, which she had never reported before. Urinary incontinence appeared occasionally, few times per year, subsiding before menstruation and intensifying after the menstrual bleeding. The patient was treated for few years conservatively with the use of muscarinic receptors antagonists achieving mediocre effect.

**Figure 5:**
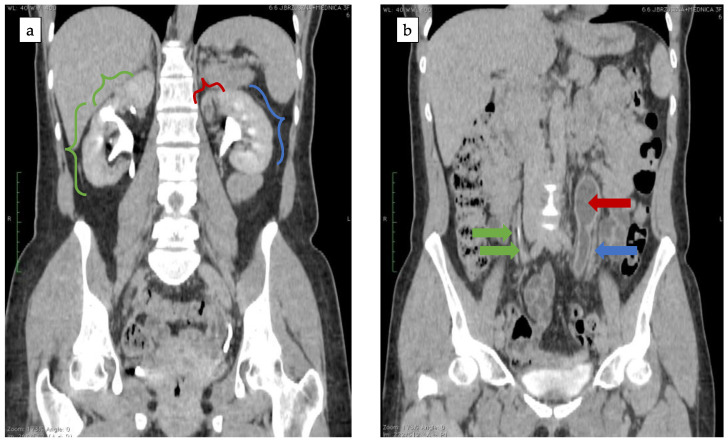
(**a**) Bilateral duplex kidney. Exertion of the contrast from both segments of right kidney (green brackets) and lower segment of the left kidney (blue bracket). No exertion of the contrast from upper moiety of the left kidney (red bracket). (**b**) Bilateral duplication of the ureter. Two contrasted ureters of the right kidney (green arrows). Contrasted ureter of the left kidney (blue arrow) and dilated, non-contrasted ureter of the upper moiety of the left kidney (red arrow).

In 2020 her symptoms worsened and became continuous without urgency nor SUI. The abdomen CT scan was repeated and showed bilateral duplex kidneys with complete duplication of ureters. The upper moiety of the left kidney showed poor function and its ureter was dilated (Figure 5).

**Figure 6:**
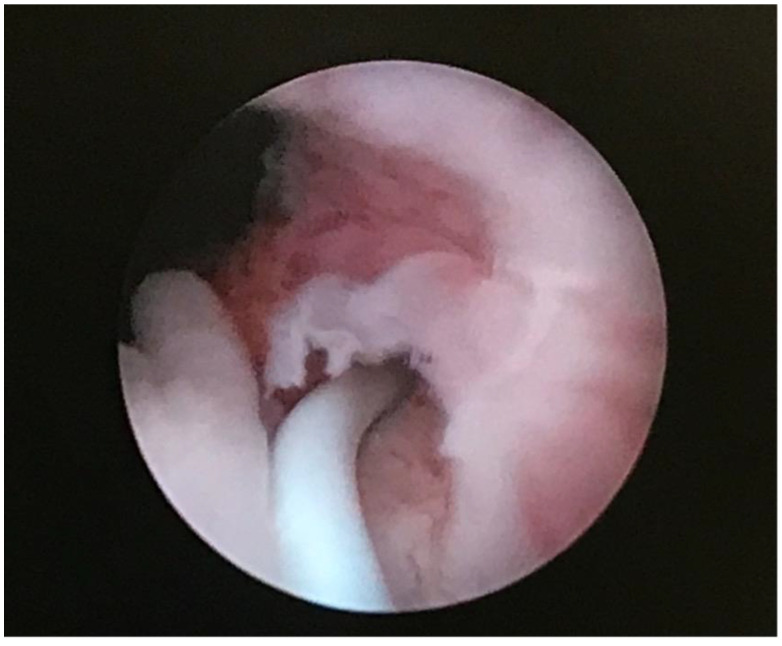
Ectopic orifice located below bladder neck probed with SJ catheter.

During diagnostic cystoscopy two orifices were found on the right side of the bladder trigon whereas only one orifice was found on the left side. The patient was diagnosed with ureteric enuresis and qualified for heminephrectomy of the upper moiety of the left kidney. In the early postoperative days patient reported minimal urinary leakage. Due to that fact a diagnostic cystoscopy was performed again. During the procedure the ectopic orifice of the dissected ectopic ureter was finally located below the bladder neck on the left side and successfully probed with SJ catheter (Figure 6). Purulent fluid was obtained. Examination of the vagina using specula was normal. SJ catheter was removed after one week and the patient was discharged home in good general condition. The patient no longer showed symptoms of urinary incontinence.

## 3. Discussion

Duplex collecting system (DCS) is one of the most common congenital renal system abnormalities. It occurs in less than 1% of the population and is more common among female population [11].

Duplex colleting systems have a variety of different phenotypes. The abnormality can be presented as a completely duplicated system (CDS)—a duplex kidney with doubled collecting systems that drain into two ureters with separate ureteral orifices and as incompletely duplicated system (IDS) with either partial ureteric duplication (Y—shaped ureter) or incomplete ureteric duplication (V—shaped ureter). H—shaped ureter, inverted Y—shaped ureter or blind ending ureter are very rare variations of ureteric fusions [6]. 

Bilateral CDS occurs 5 times less often than IDS. Duplex kidneys often coexist with other abnormalities of the urinary tract. Vesicoureteral reflux (VUR) and ureterocele are the most common anomalies associated with CDS. Other possible findings include urinary tract infections, hydronephrosis, pelvi-calyceal dilatation, cortical scarring or caliculi. Duplex kidneys are asymptomatic in most cases [12].

An ectopic ureter (EU) is a ureter that terminates outside of the trigon of the bladder. According to Weigert—Meyer law it usually concerns the upper segment ureter of duplex kidney. The orifice of an ectopic ureter may be located not only in the bladder, but also in different parts of genitourinary system such as bladder neck, urethra, ejaculatory track, vas deferens, seminal vesicles, vagina or uterus [13]. During the embryological separation/duplication of the ureteric bud, the future lower pole separates earlier and migrates superiorly and laterally. Simultaneously, the urogenital sinus migrates cranially and becomes the upper pole. As a result, the ureteric orifice of the upper pole ureter is located more medially and inferiorly on the bladder wall, whereas the ureteric orifice of the lower pole ureter is located more laterally and superiorly to the other one (Figure 3) [14].

Ureteral ectopia may manifest itself as continuous incontinence that commonly appears in early childhood. In their article, Jain et al. analyzed 9 patients with EU. According to their results, the symptoms of EU depended on the position of the ectopic ureter orifice and at 8 out of 9 patients it was urinary incontinence [15].

In many cases the localization of the ectopic ureter insertion may be problematic or even impossible and the diagnostic process often requires computed tomography with urographic phase, MRI urography or urethrocystography [16]. Finally, a cystoscopic examination may confirm the localization of the ectopic ureteral orifice [17]. Hanson et al. were able to identify ectopic ureter in all cases where they used a CT scan as a diagnostic tool. More than 20% of ectopic ureteral orifices were found in cystoscopy. They concluded that a CT scan with delayed contrast phase was highly sensitive, quick, affordable and available method for diagnosing ectopic ureters and renal systems [18].

The treatment of ureteral ectopia resulting in urinary incontinence is surgery. The most common surgical approach in case of dysplastic, poor functioning upper moiety is heminephrectomy. For kidneys with a preserved upper moiety function the ureteral reimplantation can be considered [19].

In 2019 Toia at al. published the results of their work devoted to diagnosis and outcomes of treatment of ectopic ureters in adults. They presented 10 cases (9 women and 1 man). All women complained of lifelong urinary leakage. The predominant diagnostic tool in this study was MRI. They also performed a video-urodynamic examination which additionally revealed stress urinary incontinence (SUI) in 5/9 women, urge urinary incontinence in 1/9 and mixed urinary incontinence in 2/9 women. The locations of ectopic ureteral orifice were the bladder neck in 4 cases, the subsphincteric urethra in 2 cases and the vagina in 3 cases. Further treatment depended on the kidney or its upper moiety function and included nephroureterectomy, heminephrectomy, heminephrectomy with distal ureterectomy and reimplantation of the ectopic ureter with or without bladder neck reconstruction. Additionally, other procedures like colposuspention, rectus facia sling, regular Botox injections and artificial urethral sphincter implantation have been shown to be essential in the treatment of previously diagnosed urinary incontinence. Toia et al. pointed out the role of ectopic ureter in differential diagnosis of the lifelong urinary incontinence. They recommended MRI as an examination of choice in the diagnostic process and showed that patients with ectopic ureters might require additional treatments in order to achieve continence [10].

We performed an electronic search of PubMed using a combination of keywords “urinary incontinence” and (“ectopic ureter” or “ureteral ectopia”) and “adult” limiting the results only to articles written in English over the last 10 years. We only found 20 results, most of which were single case reports. Urinary leakage, reported in most of case reports, was lifelong and related to vaginal insertion [20,21,22,23,24]. Only one article of Fichtenbaum at al. presented a case of new onset of urinary incontinence in adult life. They published a case of 61 years old female who presented with new onset urinary incontinence after undergoing organ prolapse repair surgery. During a cystoscopic evaluation the ectopic ureteral orifice was found at the bladder neck. They hypothesized that the patient might have been able to maintain continence in her young life due to the pelvic floor muscle strength. Once she got older and the muscle strength weakened, she could still maintain continence as a result of the acquired organ prolapse which created urethral kink. After the prolapse correction, the urethral kink subsided, and the urinary incontinence appeared. The patient underwent an open extra-vesical left ureteroneocystostomy and restored continence [9].

Both our patients developed urinary incontinence due to ectopic ureter in adulthood. Patient No. 1 developed syndromes of urinary incontinence as an older adult with a prolapse component. Standard procedures were performed without success. The persistence of symptoms of urinary incontinence prompted further extended diagnosis which was finally carried out a few years later. In patient No. 2 bilateral duplex collecting system with bilateral complete ureteric duplication was diagnosed earlier and ectopic ureter with ureteric enuresis was suspected and finally confirmed. We hypothesize that both patients might have been able to maintain urinary continence until adulthood because of the strength of their pelvic floor muscles and probably due to the presence of a plug of exfoliated urothelial cells occluding the distal ureteral lumen.

When the muscles weaken, either as a result of the aging process or as a result of an injury such as pregnancy and childbirth, the cell plug may be released, and symptoms of urinary incontinence appear. The severity of incontinence depends on the function of the kidney or its segment from which the ectopic ureter originates.

## 4. Conclusions

The outbreak of urinary incontinence in a course of ectopic ureter in adulthood is an extremely rarely described phenomenon, still of uncertain cause. The ectopic ureter and duplex kidney are very uncommon findings in adults and should always be taken into consideration during differential diagnosis of urinary incontinence.

## Data Availability

Not applicable.
